# Multicenter analysis of immunosuppressive medications on the risk of malignancy following adult solid organ transplantation

**DOI:** 10.3389/fonc.2023.1146002

**Published:** 2023-06-16

**Authors:** Reid Shaw, Ali R. Haque, Tyler Luu, Timothy E. O’Connor, Adam Hamidi, Jack Fitzsimons, Bianca Varda, Danny Kwon, Cody Whitcomb, Alex Gregorowicz, Gregory W. Roloff, Bradford C. Bemiss, Eric R. Kallwitz, Patrick A. Hagen, Stephanie Berg

**Affiliations:** ^1^ Department of Internal Medicine, Loyola University Medical Center, Maywood, United States; ^2^ Department of Pharmacy, Hines Veterans Affairs Hospital, Hines, United States; ^3^ Section of Hematology and Oncology, The University of Chicago, Chicago, United States; ^4^ Division of Pulmonary and Critical Care Medicine, Loyola University Medical Center, Maywood, United States; ^5^ Division of Hepatology, Loyola University Medical Center, Maywood, United States; ^6^ Division of Hematology and Oncology, Loyola University Medical Center, Maywood, United States; ^7^ Department of Medical Oncology, Lank Center for Genitourinary (GU) Dana-Farber Cancer Institute (DFCI), Harvard Medical School, Boston, MA, United States

**Keywords:** machine learning, solid organ transplant, immunosuppression, malignancy, cancer, immunosuppressive therapy

## Abstract

**Objective:**

This study aimed to assess the risk of maintenance immunosuppression on the post-transplant risk of malignancy across all solid organ transplant types.

**Methods:**

This is a retrospective cohort study from a multicenter hospital system in the United States. The electronic health record was queried from 2000 to 2021 for cases of solid organ transplant, immunosuppressive medications, and post-transplant malignancy.

**Results:**

A total of 5,591 patients, 6,142 transplanted organs, and 517 post-transplant malignancies were identified. Skin cancer was the most common type of malignancy at 52.8%, whereas liver cancer was the first malignancy to present at a median time of 351 days post-transplant. Heart and lung transplant recipients had the highest rate of malignancy, but this finding was not significant upon adjusting for immunosuppressive medications (heart HR 0.96, 95% CI 0.72 – 1.3, p = 0.88; lung HR 1.01, 95% CI 0.77 – 1.33, p = 0.94). Random forest variable importance calculations and time-dependent multivariate cox proportional hazard analysis identified an increased risk of cancer in patients receiving immunosuppressive therapy with sirolimus (HR 1.41, 95% CI 1.05 – 1.9, p = 0.04), azathioprine (HR 2.1, 95% CI 1.58 – 2.79, p < 0.001), and cyclosporine (HR 1.59, 95% CI 1.17 – 2.17, p = 0.007), while tacrolimus (HR 0.59, 95% CI 0.44 – 0.81, p < 0.001) was associated with low rates of post-transplant neoplasia.

**Conclusion:**

Our results show varying risks of immunosuppressive medications associated with the development of post-transplant malignancy, demonstrating the importance of cancer detection and surveillance strategies in solid organ transplant recipients.

## 1 Introduction

Solid organ transplant (SOT) is a curative treatment option for many patients with end-stage organ disease (1). In 2021, there were more than 40,000 organ transplants in the United States (2). Although transplant-related outcomes have significantly improved over time, rates of morbidity and mortality after successful transplantation represent areas for clinical improvement (3). One major adverse outcome after SOT is malignancy – with standardized incidence ratios of 2-4 times that of the general public (4, 5). This is due to a variety of patient, donor, transplant, cellular, and medication-related factors (6–11).

Immunosuppressive therapy is considered to be a significant risk factor in the development of malignancy following SOT as it may lead to the activation of oncogenic viruses, chronic infections, dysfunction of DNA repair, and other immune-mediated mechanisms (12–15). Prior studies have assessed the cancer risk associated with the transplantation of specific organs (16). While others have investigated immunosuppressive regimens on the risk of individual malignancies (17, 18). However, a systematic assessment of cancer development and its association with immunosuppression across all transplanted organ types has not been performed. This study aimed to assess the risk of maintenance immunosuppression on the post-transplant risk of malignancy across all SOT types. Understanding the malignancy risks associated with immunosuppressive medications across all organ transplant types may enhance the process of informed consent and better inform clinical decision-making for transplant providers.

## 2 Methods

### 2.1 Study design and data collection

This is an IRB-approved, retrospective cohort study from three academic hospitals in the greater Chicago area – Loyola University Medical Center, Gottlieb Memorial Hospital, and MacNeal Hospital. The electronic health record (EHR) software (Epic Systems; Verona, WI) was queried from January 1, 2000, to March 10, 2021. SOT and malignancies were identified using a complete list of international classification of diseases (ICD) codes from the 9^th^ and 10^th^ revisions. The date of SOT and diagnosis of malignancy was defined as the first instance an ICD code appeared in a patient’s medical record. To ease in subsequent analysis, similar ICD-9, and ICD-10 diagnoses were grouped into nominal variables.

The start and end dates of immunosuppressive medications were recorded. Immunosuppressive medications included basiliximab, belatacept, daclizumab, interferon gamma-1b, muromonab CD3, anti-thymocyte, azathioprine, cyclosporine, everolimus, mycophenolate, prednisone, sirolimus, and tacrolimus. In the case of non-continuous maintenance immunosuppressive regimens, the prior regimen was assumed to have continued until a medication change was noted in the EHR. Patient demographics, including age, sex, race, ethnicity, zip code, and preferred language were also queried from the EHR. Patients under the age of 18 were not included in this study. This study followed the Strengthening the Reporting of Observation Studies in Epidemiology (STROBE) guidelines for cohort studies (19).

### 2.2 Statistics

A two-sided t-test was calculated to assess differences in numerical variables. Chi-squared was used to assess differences in proportions. Pearson’s r was computed to assess for correlation between dichotomous variables. Loess smoothing assessed temporal trends (20). For time-to-event analysis, the development of malignancy was the event of interest. Patients were censored at the end date of the last medication, representing the last time of contact with the study center, or at death. For each patient, every transplant and new malignancy diagnosis represented a new observation within the data frame. Gray’s test was calculated for competing risk analysis (21). Multivariable regression was performed using Cox proportional hazard with time to malignancy as the dependent variable. Independent variables included medications, age at transplant, race, sex, and transplanted organ. Test for proportional hazard assumption was performed on all independent variables (22). Maintenance immunosuppressive medications were coded as time-dependent covariates (23, 24). Changes in medication dosages were not modeled. Statistical significance was defined as p-value < 0.05, and, when appropriate, a Bonferroni correction was applied (25).

### 2.3 Machine learning

A random forest model was used to assess variable importance in predicting post-transplant malignancy (26). The total number of immunosuppressive medications was calculated as the sum of induction and maintenance agents before the event or censoring. Categorical variables with a frequency of less than 10% were grouped into an “other” category. Nominal variables were one-hot encoded and numerical variables were normalized to have a standard deviation of one with a mean of zero. The complete dataset was then bootstrap resampled 10 times and stratified by the outcome. A random forest model was then fit across a variety of hyperparameters within a Latin hypercube of size 25 (27). Gini impurity values were calculated to provide a robust assessment of variable importance (28).

### 2.4 Analysis code

All analysis was performed with R programming language v.4.0.3 (29). using the ‘tidyverse’ (30), ‘tidymodels’ (31), ‘lubridate’ (32), ‘funneljoin’ (33), ‘ggridges’ (34), ‘surival’ (35), ‘janitor’ (36), ‘wesanderson’ (37), ‘ochRe’ (38), ‘scales’ (39), ‘vip’ (40), ‘resample’ (41), ‘corrplot’ (42), ‘timereg’ (43), ‘patchwork’ (44), and ‘tidycmprsk’ (45) packages. The complete analysis code is available at: https://github.com/reidshaw/sot_malignancy.

## 3 Results

### 3.1 Cohort characteristics

**Figure 1:**
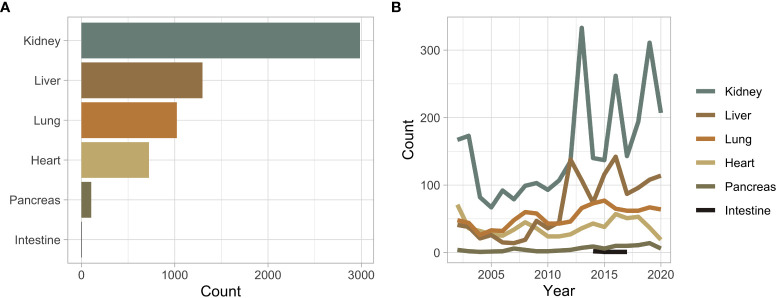
**(A)** Bar plot of the overall number of solid organ transplant recipients stratified by organ type. **(B)** Solid organ transplant recipients across time stratified by organ type.

Overall, 5,591 unique patients received a SOT during the queried period, comprising six different organ types, and 6,142 transplanted organs (**Figure 1A**). Throughout the study period, the annual number of SOT increased (**Figure 1B**). The median time to event or censor was 1,903 days. Kidney transplants (n = 2,986) were the most common, followed by liver (n = 1,298), lung (n = 1,024), heart (n = 723), pancreas (n = 106), and intestine (n = 5) (**Figure 1A**). The median age of a transplant recipient was 54 years with a range of 18-91 years (**Supplementary Figure 1A**). There were 5,093 people who received one SOT, 448 received two organs, 47 received three organs, and three received four organs (**Supplementary Figure 1B**). Sixty-four percent of transplant recipients self-identified as White, while the next most common racial demographic was Black at 17% (**Supplementary Figure 1C**). White and Asian transplant recipients were the oldest demographic to receive an organ at a median age of 56 years (**Supplementary Figure 1A**). These two groups were statistically older than other minority groups, including Black (52 years), Hispanic (50 years), and multi-racial (40 years) (**Supplementary Figure 1A**). The median age was highest in lung (58 years), heart (58 years), and liver (59 years) recipients (**Supplementary Figure 1D**). Overall, 39% of transplant recipients were women. Except for intestine transplant recipients, SOT was more common in men than in women (**Supplementary Figure 1E**). Loess smoothing showed a trend towards women becoming less likely to receive SOT when compared to men (**Supplementary Figure 1F**).

### 3.2 Malignancy statistics

**Figure 2:**
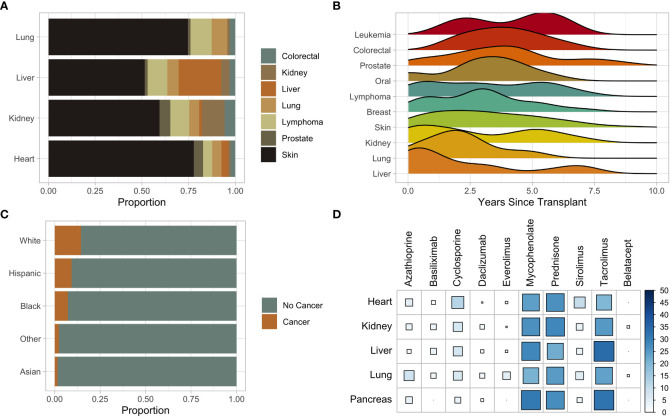
**(A)** Stacked bar plot of subsequent malignancies by solid organ transplant type. **(B)** Ridge plot of subsequent malignancies from time of transplant. **(C)** Stacked bar plot of the proportion of subsequent malignancies stratified by race. **(D)** Bubble plot of immunosuppressive medications stratified by solid organ transplant. Color and size indicate the percent in which the immunosuppression was used in the transplant recipient.

There were 517 (8.25%) post-SOT malignancies identified (**Supplementary Table 1**). Skin cancer (n = 273) was the predominant malignancy, followed by lymphoma (n = 40), and kidney (n = 30) (**Figure 2A**). In liver and kidney transplant recipients, post-transplant liver and kidney malignancies represent the largest proportional increase from baseline rates, respectively (**Figure 2A**). Liver cancer was the earliest malignancy to present following SOT at an average of 351 days (**Figure 2B**). The median presentation of skin cancer was 1,073 days, breast cancer at 1,109 days, and lymphoma at 1,123 days (**Figure 2B**). Leukemia (n = 12) was the malignancy with the longest post-transplant latency time to presentation at 1,735 days (**Figure 2B**). Fourteen percent of White transplant recipients were diagnosed with a post-SOT malignancy; Hispanic and Black individuals were diagnosed at 9% and 7%, respectively (**Figure 2C**). Of the 249 Asian patients who received SOT, only four (1.6%) developed a post-transplant malignancy (**Figure 2C**). Other than lung transplant recipients (p = 0.048), no other SOT recipient group had a significant difference in post-transplant malignancies based on the age of transplantation.

### 3.3 Immunosuppression and risk of malignancy

**Figure 3:**
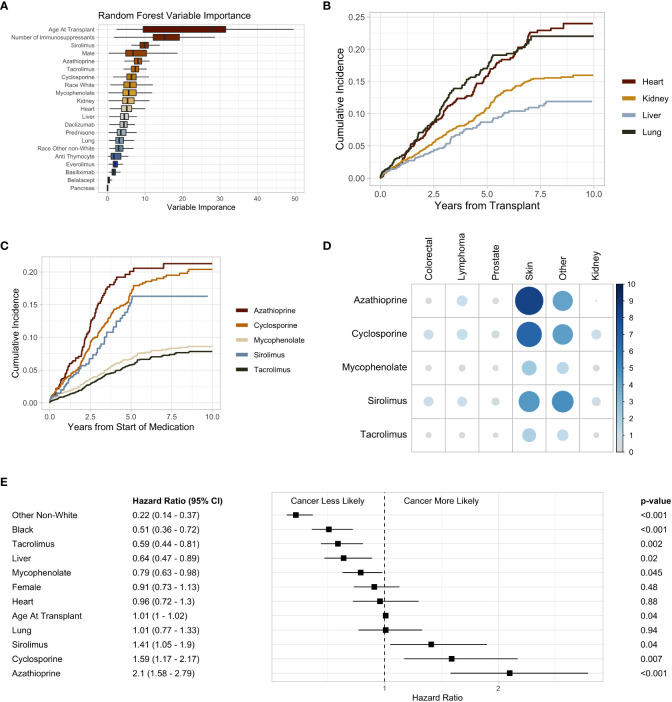
**(A)** Box plot of random forest Gini impurity values indicating variable importance in predicting malignancy following solid organ transplant. **(B)** Subsequent malignancy cumulative incidence plot stratified by organ type. **(C)** Subsequent malignancy cumulative incidence plot stratified by immunosuppressive medication. **(D)** Heatmap of immunosuppression and post-transplant malignancy. The size and shade of blue indicate percent of individuals to develop a malignancy. **(E)** Forest plot of the multivariate Cox proportional hazard ratios. Black and Other Non-White hazard ratios are in comparison to White. Heart, liver, and lung are in comparison to kidney transplant recipients. P-values are adjusted with a Bonferroni correction factor.

The most common immunosuppressive medications used were mycophenolate and tacrolimus (**Supplementary Figure 2A**). The use of these medications increased throughout the study duration (**Supplementary Figure 2B**). Cyclosporine and sirolimus were most commonly utilized in heart transplant recipients, at 12% and 10%, respectively (**Figure 2D**). Azathioprine was most commonly used in lung transplant recipients at 33%, and 91% of liver transplant recipients received tacrolimus (**Figure 2D**). Tacrolimus and mycophenolate (phi coefficient 0.36) were the drugs most commonly used in the same patients, whereas tacrolimus and cyclosporine (phi coefficient -0.36) were infrequently used together (**Supplementary Figure 3**). Upon fitting a random forest machine learning (ML) model to assess variable importance on the development of malignancy, age at transplantation was the most predictive variable followed by the total number of immunosuppressive medications a patient received (**Figure 3A**). The immunosuppressive medication with the strongest association with post-SOT malignancy was sirolimus, followed by azathioprine and tacrolimus (**Figure 3A**).

Heart and lung transplant recipients had a high cumulative incidence of post-SOT malignancy in our study (**Figure 3B**). Within 30 months post-transplant, 8.5% of heart and 8.7% of lung transplant recipients received a subsequent diagnosis of cancer (**Supplementary Figure 4**). At 60 months, this number increased to 16% and 17% for heart and lung transplant recipients, respectively (**Supplementary Figure 4**). The cumulative incidence of post-transplant malignancy was greatest in patients who received sirolimus, azathioprine, and cyclosporine, whereas mycophenolate and tacrolimus had a relatively lower incidence (**Figure 3C**). Azathioprine (8.4%) and cyclosporine (6.7%) had the highest rates of skin cancer (**Figure 3D**). Whereas, mycophenolate (0.45%) and tacrolimus (0.36%) had the lowest rates of lymphoma (**Figure 3D**).

Multivariate Cox regression analysis demonstrated an increased risk of malignancy with azathioprine (HR 2.1, 95% CI 1.58 – 2.79, p < 0.001), cyclosporine (HR 1.59, 95% CI 1.17 – 2.17, p = 0.007), and sirolimus (HR 1.41, 95% CI 1.05 – 1.9, p = 0.04). Tacrolimus (HR 0.59, 95% CI 0.44 – 0.81, p < 0.001) was associated with a lower risk of post-transplant malignancy (**Figure 3E**). When compared to kidney transplant recipients, liver recipients (HR 0.42, 95% CI 0.47 – 0.89, p = 0.02) were less likely to develop a malignancy (**Figure 3E**). There was no significant difference in the risk of post-transplant malignancy between men and women or the age at which someone received a SOT (**Figure 3E**). Black (HR 0.51, 95% CI 0.36 – 0.72, p < 0.001) and other non-White (HR 0.22, 95% CI 0.14 – 0.37, p < 0.001) SOT recipients were less likely to develop a malignancy than White individuals (**Figure 3E**). However, there was no difference in the rates of non-cutaneous malignancies between Black and White transplant recipients (**Supplementary Figure 5**).

## 4 Discussion

In this multicenter cohort study of 6,142 transplanted organs followed for a median follow-up of 1,903 days, we found significant variations in the risk of malignancy amongst maintenance immunosuppressive medications. Our study highlights several important observations about longitudinal immunosuppression and the risk of malignancy across all types of SOT. The risk of malignancy did not appear to be dependent on the transplanted organ. Additionally, our statistical modeling and machine learning algorithms demonstrate that the risk of subsequent malignancy was segregated by immunosuppressive agents. We observed the highest rate of subsequent malignancy in patients who received azathioprine, followed by cyclosporine and sirolimus. Tacrolimus was associated with the lowest risk of malignancy among the immunosuppressant agents considered.

Prior research has identified higher rates of post-SOT malignancy in heart and lung recipients compared to those receiving kidney or liver transplants (46, 47). However, upon adjusting for immunosuppressive medications, we found no difference in the rates of malignancy between SOT groups, suggesting a potential contribution to neoplasia imparted by the biological effects of the post-transplant immunosuppression (48, 49).

Due to tissue rejection and medication toxicity, a patient’s immunosuppressive regimen often changes (50). By treating immunosuppressive medications as time-varying covariates, we temporally assessed the risk of immunosuppression (24). In our study, azathioprine, cyclosporine, and sirolimus were associated with the highest risk of cancer development. Azathioprine, an antagonist of purine metabolism, has long been associated with the development of cancer in SOT, inflammatory bowel disease, multiple sclerosis, and rheumatoid arthritis (51–55). Within our study, we observed the highest rates of skin cancer with the use of azathioprine. In addition to the direct immunosuppressive effects of azathioprine, this finding is likely magnified due to the role azathioprine plays in DNA synthesis and repair – a key mechanism in the pathogenesis of skin cancer (14, 56).

Consistent with previous studies, cyclosporine was also associated with high rates of skin cancer (57). This finding is partially due to the role cyclosporine plays in the inhibition of ultraviolet-B-induced apoptosis and DNA repair (58). However, higher rates of kidney cancer were also seen with cyclosporine use. Previous studies have demonstrated a cyclosporine dose-dependent risk of malignancy in kidney transplant recipients (59). The higher rates of kidney cancer that we observe may be due to the induction of transforming growth factor-beta by cyclosporine, increasing cellular proliferation, and decreasing differentiation (60–62).

The increased risk of cancer with sirolimus was unexpected as prior studies have generally demonstrated decreased risk with sirolimus use (63–66). However, in concordance with a randomized trial that investigated the risk of malignancy in kidney transplant recipients treated with sirolimus, we found a decreased risk of the development of skin cancer with sirolimus use across all SOT recipients (67, 68). In prior studies of kidney and heart transplant recipients, the transition from a calcineurin inhibitor to sirolimus was associated with a lower risk of malignancy (69, 70), likely due in part to the role mTOR plays in cell proliferation (71). In liver transplant recipients, cumulative exposure to tacrolimus increased the risk of cancer (72). This finding was not unsurprising and not in opposition to our data as we did not assess serum levels of tacrolimus. However, our data demonstrated a lower risk of cancer in individuals who received tacrolimus compared to other immunosuppressive medications.

ML algorithms are now being applied to a variety of SOT research questions (73). To our knowledge, our study represents the first time that a ML model has been used to assess variable importance in determining which SOT recipients developed a malignancy. Random forest classification, a form of decision trees, is a highly flexible, interpretable, and accurate method of estimating non-linear relationships – an area where traditional statistics struggle (74). ML models can be applied to feature selection and outcome prediction. In contrast to traditional statistical methods used in this analysis, our ML model identified age at transplant as a highly predictive marker of post-transplant malignancy diagnosis. However, not all results were dissimilar, as the ML model also identified sirolimus, tacrolimus, azathioprine, and cyclosporine as highly predictive variables.

We consider the internal validity of this investigation to be high as our data are consistent with other landmark studies. For example, our data demonstrated that the majority of liver cancer diagnoses occur within the first year of transplantation – likely due to hepatocellular carcinoma recurrence following transplantation (5, 75). In addition, skin cancer was the most common post-transplant malignancy in our cohort, followed by lymphoma and kidney cancer (46). However, there are several limitations to this study. First, there are likely discrepancies in data entry, collection, and classification that may exist as this was a retrospective cohort study based on ICD codes. We chose to omit a formal control group with the calculation of standardized incidence ratios, focusing instead on the comparison between medications. The rates of malignancy are likely underestimated as we included individuals who received transplants up to the study endpoint. In addition, patients may be lost to follow-up, and subsequent cancer diagnoses may be made outside of the queried hospital system. In addition, the centers in this study are located in the greater Chicago area and thus may not represent results from distinct geographical regions across the United States or in other countries. Compared to national database studies, our sample size is small, preventing the stratification of individual malignancies and association with particular immunosuppression regimens. Furthermore, we did not account for the dose of immunosuppression or blood plasma level of these medications. We also did not control for any pre-transplant-related criteria, including organ ischemic time, viral studies, donor information, screening tests, or education level. This may confound some of the findings that we attribute to sociodemographic factors and immunosuppressive medications. Lastly, our findings do not establish causality but provide further data to underscore the importance of cancer detection and surveillance strategies in organ transplant recipients.

## Data availability statement

The raw data supporting the conclusions of this article will be made available by the authors, without undue reservation.

## Ethics statement

The studies involving human participants were reviewed and approved by Loyola University IRB. Written informed consent for participation was not required for this study in accordance with the national legislation and the institutional requirements.

## Author contributions

Study concept and design: RS, EK, SB. Acquisition of data: RS, AH, TL, JF, AH, TO’C, BV, DK, CW, AG, GR, BB, EK, PH, SB. Statistical analysis: RS. Drafting of the manuscript: RS, GR. All authors contributed to the article and approved the submitted version.

## Supplementary material

Supplementary Figure 1
**(A)** Box plots of the age of solid organ transplant recipients stratified by race **(B)** Bar plot of the number of solid organ transplant recipients per person. **(C)** Bar plot of the number of solid organ transplant recipients stratified by race. **(D)** Box plots of the age of solid organ transplant recipients grouped by organ type. **(E)** Grouped bar plot of solid organ transplant types stratified by sex. Male is denoted by ‘M’ and Female is denoted by ‘F.’ **(F)** Dot plot showing the percentage of women who received a solid organ transplant across the study period. A Loess-smoothed line is overlayed.

Supplementary Figure 2
**(A)** Bar plot of the most common immunosuppressants ordered by the total number. **(B)** Line plot of the use of individual maintenance immunosuppression medications throughout time.

Supplementary Figure 3 Pearson correlation matrix of immunosuppressive medications. A positive correlation is indicated by blue and a negative correlation is indicated by red. Non-significant correlations are left blank. Significance is defined as p-value < 0.05.

Supplementary Figure 4 Subsequent malignancy cumulative incidence at 2.5 years and 5 years stratified by organ type. 95% confidence intervals are in brackets.

Supplementary Figure 5 Cumulative incidence plot of non-cutaneous cancers stratified by race.

Supplementary Table 1 CSV file of ICD-9, and ICD-10 diagnoses with associated grouping into nominal malignancy types.
