# Supplementary material for: Multicenter analysis of immunosuppressive medications on the risk of malignancy following adult solid organ transplantation
Source: Front Oncol. 2023 Jun 16;13:1146002. doi: 10.3389/fonc.2023.1146002 (PMC10313202; doi:10.3389/fonc.2023.1146002)
Supplement: Supplementary Figure 1 — (A) Box plots of the age of solid organ transplant recipients stratified by race (B) Bar plot of the number of solid organ transplant recipients per person. (C) Bar plot of the number of solid organ transplant recipients stratified by race. (D) Box plots of the age of solid organ transplant recipients grouped by organ type. (E) Grouped bar plot of solid organ transplant types stratified by sex. Male is denoted by ‘M’ and Female is denoted by ‘F.’ (F) Dot plot showing the percentage of women who received a solid organ transplant across the study period. A Loess-smoothed line is overlayed. [file Image_1.pdf]

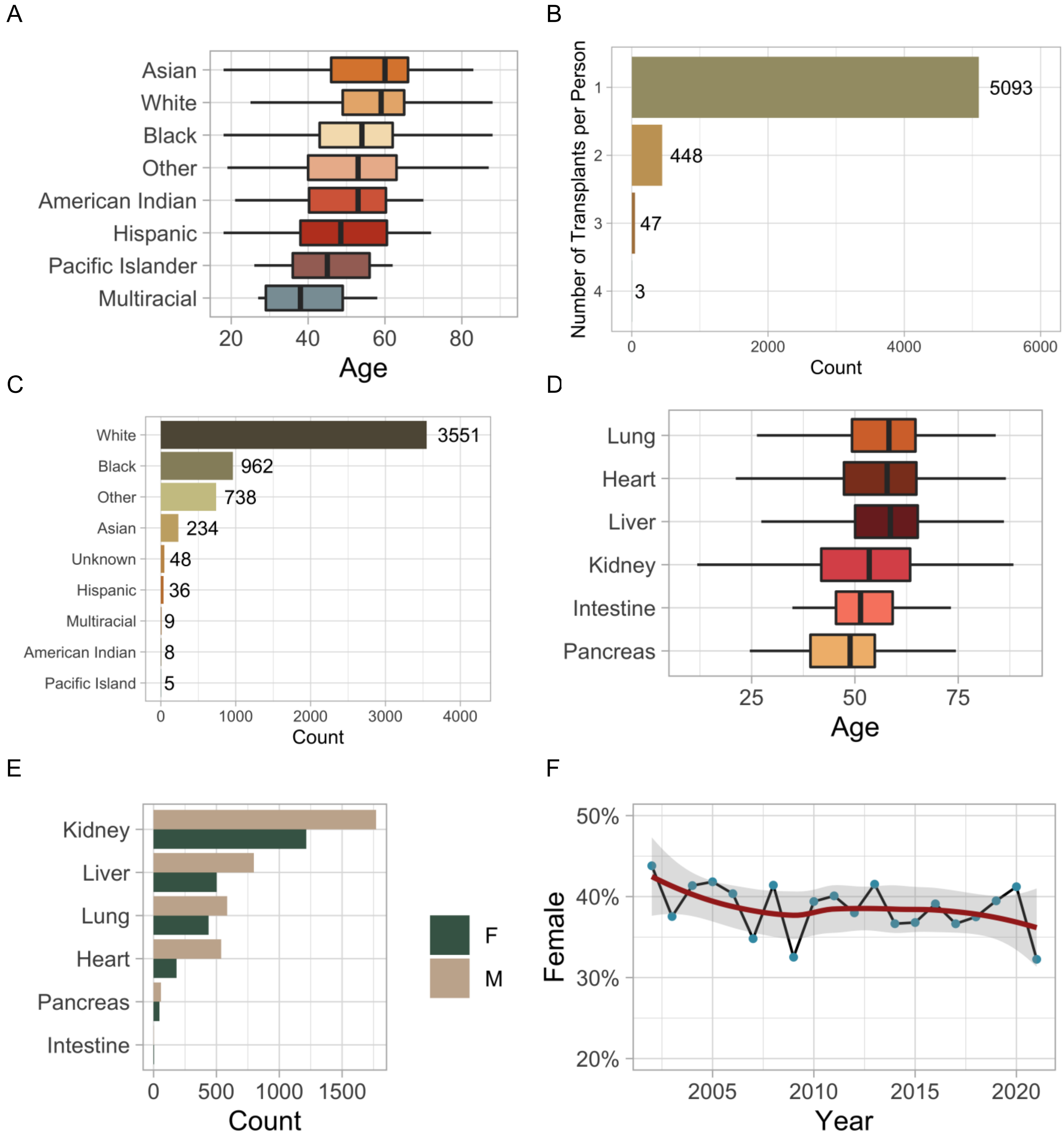

A
Asian
White
Black
Other
American Indian
Hispanic
Pacific Islander
Multiracial
20
40
60
80
Age
B
Number of Transplants per Person
1
2
3
4
5093
448
47
3
0
2000
4000
6000
Count
C
White
Black
Other
Asian
Unknown
Hispanic
Multiracial
American Indian
Pacific Island
3551
962
738
234
48
36
9
8
5
0
1000
2000
3000
4000
Count
D
Lung
Heart
Liver
Kidney
Intestine
Pancreas
25
50
75
Age
E
Kidney
Liver
Lung
Heart
Pancreas
Intestine
F
M
0
500
1000
1500
Count
F
Female
50%
40%
30%
20%
2005
2010
2015
2020
Year

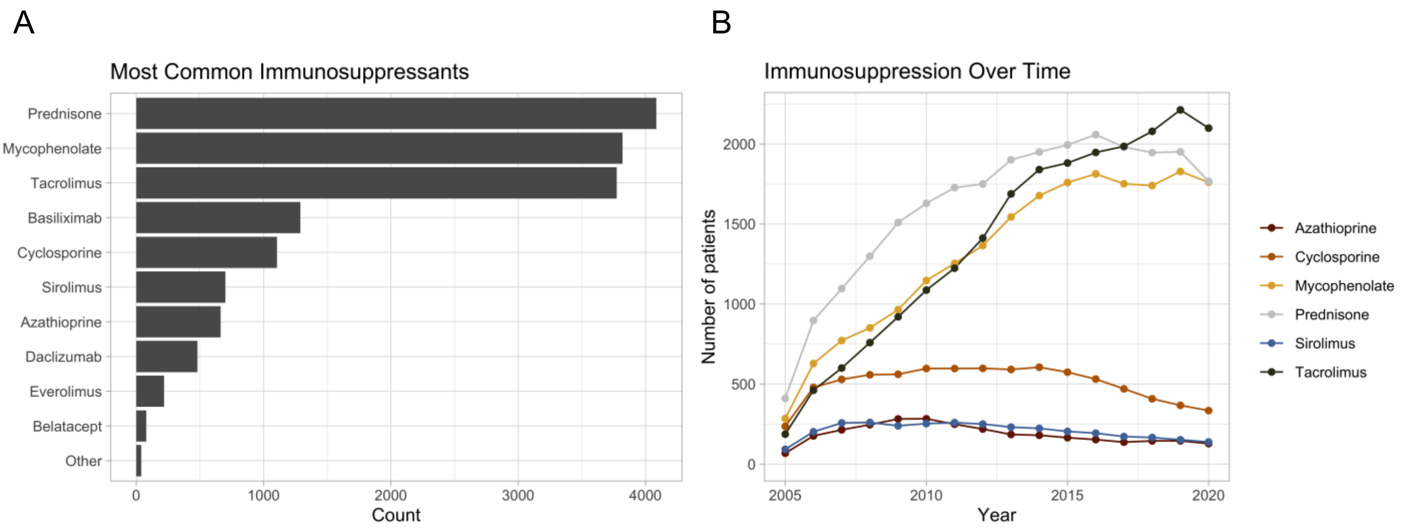
A
Most Common Immunosuppressants
Prednisone
Mycophenolate
Tacrolimus
Basiliximab
Cyclosporine
Sirolimus
Azathioprine
Daclizumab
Everolimus
Belatacept
Other
0
1000
2000
3000
4000
Count
B
Immunosuppression Over Time
Number of patients
0
500
1000
1500
2000
2005
2010
2015
2020
Year
Azathioprine
Cyclosporine
Mycophenolate
Prednisone
Sirolimus
Tacrolimus

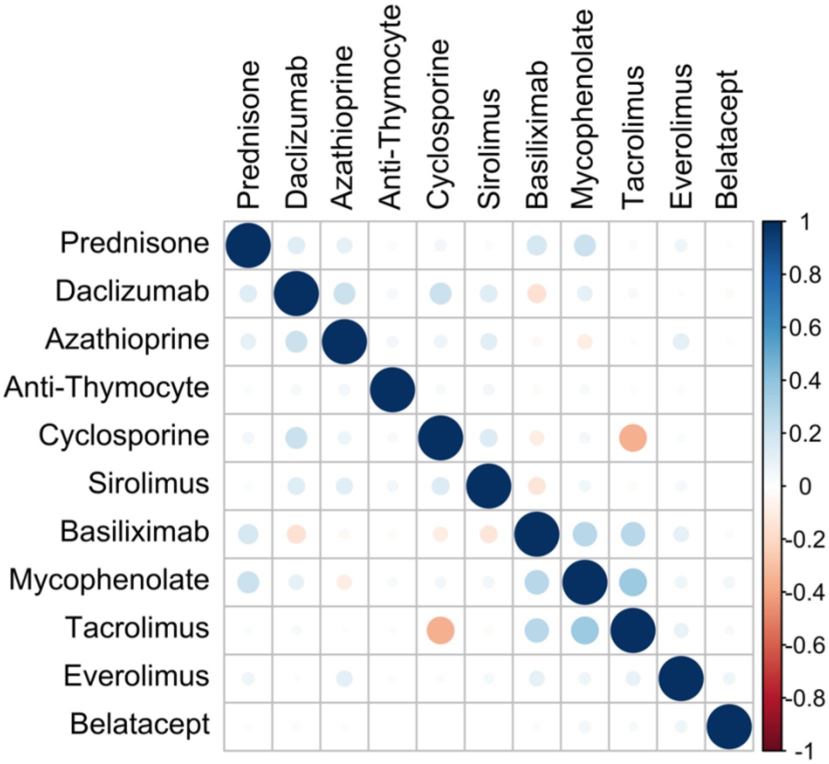

Prednisone
Daclizumab
Azathioprine
Anti-Thymocyte
Cyclosporine
Sirolimus
Basiliximab
Mycophenolate
Tacrolimus
Everolimus
Belatacept
1
0.8
0.6
0.4
0.2
0
-0.2
-0.4
-0.6
-0.8
-1

Characteristic	Year 2.5	Year 5	p-value[1]
Transplant			<0.001
Heart	8.5% (6.5%, 11%)	16% (13%, 19%)	
Intestine	0.00% (—%, —%)	0.00% (—%, —%)	
Kidney	5.6% (4.7%, 6.6%)	11% (9.5%, 12%)	
Liver	4.4% (3.2%, 5.8%)	8.7% (6.8%, 11%)	
Lung	8.7% (6.9%, 11%)	17% (14%, 20%)	
Pancreas	1.7% (0.14%, 8.0%)	1.7% (0.14%, 8.0%)	

[1] Gray's Test

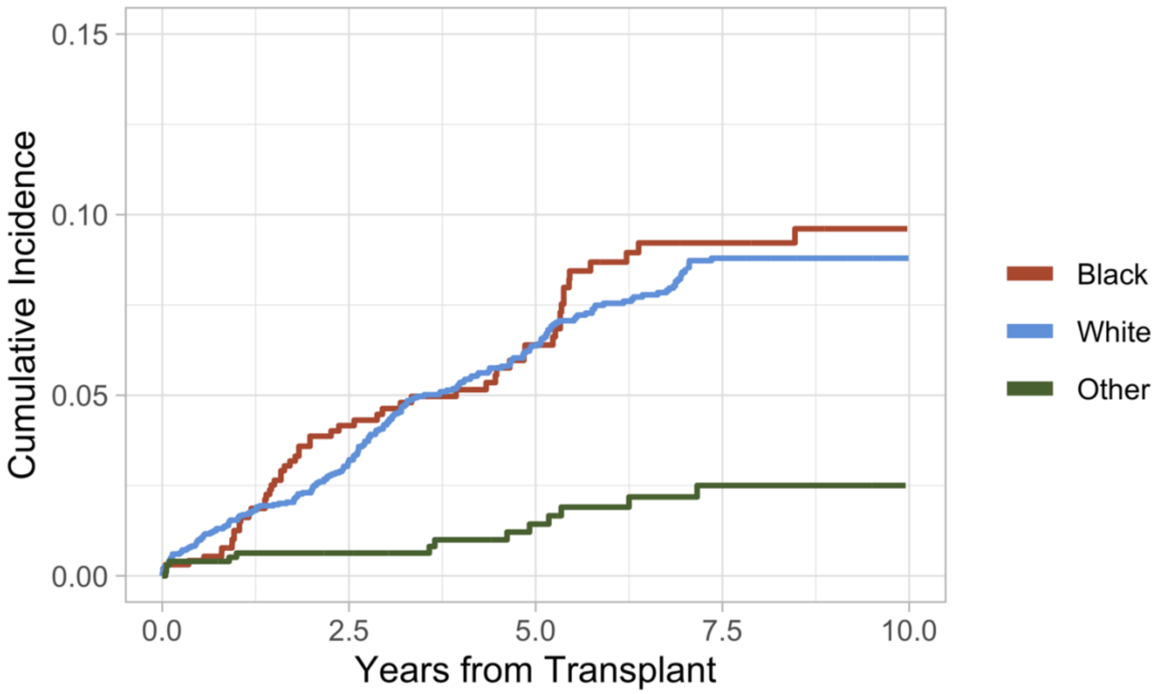

Cumulative Incidence
0.15
0.10
0.05
0.00
0.0
2.5
5.0
7.5
10.0
Years from Transplant
Black
White
Other
